# Phase I Clinical Trial Using [^99m^Tc]Tc-1-thio-D-glucose for Diagnosis of Lymphoma Patients

**DOI:** 10.3390/pharmaceutics14061274

**Published:** 2022-06-15

**Authors:** Vladimir Chernov, Ekaterina Dudnikova, Roman Zelchan, Anna Medvedeva, Anstasiya Rybina, Olga Bragina, Viktor Goldberg, Albina Muravleva, Jens Sörensen, Vladimir Tolmachev

**Affiliations:** 1 Department of Nuclear Medicine, Cancer Research Institute, Tomsk National Research Medical Center, Russian Academy of Sciences, 634050 Tomsk, Russia; chernov@tnimc.ru (V.C.); zelchanrv@onco.tnimc.ru (R.Z.); medvedeva@tnimc.ru (A.M.); pankovaan@mail.ru (A.R.); rungis@mail.ru (O.B.); 2 Research Centrum for Oncotheranostics, Research School of Chemistry and Applied Biomedical Sciences, Tomsk Polytechnic University, 634050 Tomsk, Russia; 3 Department of Cancer Chemotherapy, Cancer Research Institute, Tomsk National Research Medical Center, Russian Academy of Sciences, 634050 Tomsk, Russia; ekaterina.dudnikova@list.ru (E.D.); goldbergve@mail.ru (V.G.); albina_danilova7487@mail.ru (A.M.); 4 Radiology and Nuclear Medicine, Department of Surgical Sciences, Uppsala University, 751 83 Uppsala, Sweden; jens.sorensen@pet.uu.se; 5 Department of Immunology, Genetics and Pathology, Uppsala University, 751 83 Uppsala, Sweden

**Keywords:** [^99m^Tc]Tc-1-thio-D-glucose, single-photon emission computed tomography, lymphoma, Hodgkin’s lymphoma, non-Hodgkin’s lymphomas

## Abstract

Similar to [^18^F]-FDG, [^99m^Tc]Tc-1-thio-D-glucose ([^99m^Tc]Tc-TG) also binds to GLUT receptors. The aim of this Phase I study was to evaluate the safety, biodistribution and dosimetry of [^99m^Tc]Tc-TG. Twelve lymphoma patients were injected with 729 ± 102 MBq [^99m^Tc]Tc-TG. Whole-body planar imaging was performed in 10 patients at 2, 4, 6 and 24 h after injection. In all 12 patients, SPECT/CT (at 2 h) and SPECT (at 4 and 6 h) imaging was performed. Vital signs and possible side effects were monitored during imaging and up to 7 days after injection. [^99m^Tc]Tc-TG injections were well-tolerated and no side effects or alterations in blood and urine analyses data were observed. The highest absorbed dose was in the kidneys and urinary bladder wall, followed by the adrenals, prostate, bone marrow, lungs, myocardium, ovaries, uterus, liver and gall bladder wall. [^99m^Tc]Tc-TG SPECT/CT revealed foci of high activity uptake in the lymph nodes of all nine patients with known nodal lesions. Extranodal lesions were detected in all nine cases. In one patient, a lesion in the humerus head, which was not detected by CT, was visualized using [^99m^Tc]Tc-TG. Potentially, [^99m^Tc]Tc-TG can be considered as an additional diagnostic method for imaging GLUT receptors in lymphoma patients.

## 1. Introduction

**Figure 1:**
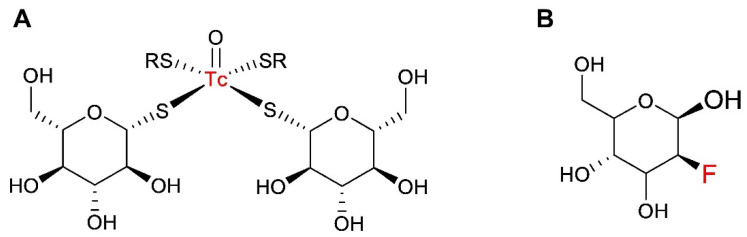
Schematic representation of structures of [^99m^Tc]Tc-TG (**A**) and [^18^F]-FDG (**B**).

Positron emission tomography (PET), and more recently PET/CT, using [^18^F]-2-fluoro-2-deoxy-D-glucose ([^18^F]-FDG), is the most sensitive and specific imaging technique currently available for patients with lymphoma. Numerous studies indicate the utility of [^18^F]-FDG PET/CT for staging lymphomas, predicting their response to therapy and evaluating their treatment effectiveness [1,2]. Despite the advantages of this technology for patients’ management, the use of PET in many developing countries is limited by the high cost of studies and low number of PET/CT facilities. However, SPECT scanners are installed in many hospitals around the world and, therefore, the use of tracers labeled with gamma-emitting nuclides for the imaging of hypermetabolic lesions might be a solution for these countries. Several glucose analogues have been preclinically evaluated for the SPECT imaging of tumor metabolisms [3,4,5,6,7], and [^99m^Tc]Tc-1-thio-D-glucose ([^99m^Tc]Tc-TG) is one of such tracers (Figure 1). The mechanism of how [^99m^Tc]Tc-TG binds to malignant cells was elucidated using colorectal carcinoma (HCT-116) and human lung adenocarcinoma (A549) cells [4]. The [^99m^Tc]Tc-TG and [^18^F]-FDG accumulation level in HCT-116 cells was very close; however, the [^18^F]-FDG uptake in the A549 cell line was almost double that of [^99m^Tc]Tc-TG. The cellular accumulation of both [^99m^Tc]Tc-TG and [^18^F]-FDG decreased with an increase of the glucose competitor concentration, and was increased in the presence of insulin. These observations implied that both [^18^F]-FDG and [^99m^Tc]Tc-TG bound to cells with the participation of glucose transport proteins. Furthermore, cellular uptake of both [^18^F]-FDG and [^99m^Tc]Tc-TG was reduced in the presence of cytochalasin B, which blocks the GLUT1-5 channels. On the contrary, no reduction of cellular uptake was observed for either [^99m^Tc]Tc-TG or [^18^F]-FDG when SGLT1-3 was blocked by phloretin, suggesting that these transporters are not involved in the uptake process [4]. This lead to the conclusion that these agents are taken up by the cells mainly via GLUT transporters, which was in agreement with the data demonstrating a substantial overexpression of GLUT1 in the tested cell lines [8,9,10]. Importantly, [^99m^Tc]Tc-TG accumulates predominantly in cell membranes, whereas [^18^F]-FDG is predominantly localized in the cytoplasm. This feature was explained by the large size of the [^99m^Tc]Tc-TG molecule, which might hamper its transport into cells, and the tracer remains attached to membrane-bound transporter proteins [4]. Overall, this study suggested that [^99m^Tc]Tc-TG could be used for the mapping of sites with elevated GLUT expression, including malignant tumors.

In preparation for clinical studies, a single-vial kit for labeling 1-thio-D-glucose and the analytical methods for characterizing the labeled product were developed [11]. Further preclinical studies demonstrated that [^99m^Tc]Tc-TG does not have any acute or cumulative toxicity and is not allergenic. Accumulation of [^99m^Tc]Tc-TG in tumor cells, including the RMA lymphoma cell line, was demonstrated in vivo and in vitro [11,12].

There were three primary objectives of this first-in-human study: firstly, to obtain initial information concerning the safety and tolerability of [^99m^Tc]Tc-TG after a single intravenous injection; secondly, to assess the distribution of [^99m^Tc]Tc-TG in normal tissues and in tumors over time; and thirdly, to evaluate the dosimetry of [^99m^Tc]Tc-TG.

The secondary objective was to evaluate the possibility of using [^99m^Tc]Tc-TG SPECT/CT for visualizing GLUT expression in the nodal and extranodal lesions of lymphoma patients.

## 2. Materials and Methods

### 2.1. Patients

This was a prospective, open-label, non-randomized diagnostic study in patients with untreated lymphoma (ClinicalTrials.gov Identifier: NCT04292626). The protocol was approved by the Scientific Council of Cancer Research Institute and Board of Medical Ethics, Tomsk National Research Medical Center of the Russian Academy of Sciences. All subjects signed written informed consent forms. 

Patients (18–70 years) with lymphoma were eligible and accepted based on the following inclusion criteria: (1) clinical and radiological diagnosis of Hodgkin lymphoma and non-Hodgkin lymphoma with immunohistologic verification; (2) volumetrically quantifiable tumor lesions on the CT or ultrasound, with at least one lesion >1.0 cm in the greatest diameter; (3) hematological, liver and renal function test results within the normal limits; (4) negative pregnancy test for female patients of childbearing potential; and (5) capability to undergo the diagnostic investigations planned in the study.

Patients with lymphoma were not accepted based one or more of the following exclusion criteria: (1) a previous diagnosis with an autoimmune disease; (2) an active infection or history of severe infection; (3) known to be HIV positive or have a chronically active hepatitis B or C infection; or (4) had been administered other investigational medicinal products.

Twelve patients (four male and eight female) were enrolled (Table 1, Figure S1) and, as a local standard of care, chest and abdomen CT (Siemens Somatom Emotions 16 ECO) and ultrasound (GE LOGIQ E9) imaging was performed along with biopsy sampling for all patients. Biopsy samples of nodal and extranodal lesions were collected and the diagnosis was verified by immunohistochemistry (IHC).

### 2.2. Radiopharmaceutical 

Labeling of [^99m^Tc]Tc-TG was performed in aseptic conditions using a single-vial kit described earlier [11]. Briefly, kits containing 2.5 mg of the glucose derivative, 0.21 mg of tin chloride dihydrate and 0.5 mg of ascorbic acid each were prepared in aseptic conditions by freeze-drying (−50 °C, 24 h). For labelling with ^99m^Tc, a kit was reconstituted in a generator eluate (4 mL) containing [^99m^Tc] sodium pertechnetate, incubated for 30 min at room temperature and passed through a sterile filter. The radiochemical yield was determined using radio-ITLC (instant thin-layer chromatography) using silica-gel strips (TLC-SG Sorbfil, Imid, Krasnodar, Russia). To determine the presence of free [^99m^Tc] pertechnetate, the strips were developed using acetone (Rf for [^99m^Tc]Tc-TG = 0; Rf for [^99m^Tc] pertechnetate = 0.9–1.0). To determine the presence of technetium radiocolloid, a mixture ethanol: NH_4_OH: water (2:5:5) was used (Rf for [^99m^Tc]Tc-radiocolloid = 0; Rf for other forms of ^99m^Tc = 0.9–1.0). The ITLC was cross-validated using HPLC using ZORBAX NH2 column (4.6 × 250 mm, 5 μm, Agilent, Santa Clara, CA, USA).

It was demonstrated during the tracer development that the labelled compound is stable under challenge with a 1000-fold excess of L-cysteine during 4 h (no measurable release of free ^99m^Tc).

For a release of the clinical batch, a test labelling was performed using 10 kit vials. [^99m^Tc]Tc-TG was tested according to State Pharmacopoeia of the Russian Federation, XIII ed. (SP XIII) for the authenticity of all reagents, as well as for pH, volume activity, radiochemical purity, bacterial endotoxin content (LAL test) and sterility using thioglycollate fluid medium and Sabouraud Dextrose Broth. [^99m^Tc]Tc-TG met all Pharmacopoeia requirements, was sterile and pyrogen-free.

During the clinical trial, the radiochemical purity of [^99m^Tc]Tc-TG was 97 ± 1%.

### 2.3. Imaging Protocol

The [^99m^Tc]Tc-TG, in a dose of 729 ± 102 MBq, was injected as an intravenous bolus. A Siemens Symbia Intevo Bold scanner was used for imaging. A high-resolution low-energy collimator was used to images acquisition. Anterior and posterior whole-body planar imaging (at a scan speed of 12  cm/min, 1024 × 256 pixel matrix) was carried out in patients 1–10 at 2, 4, 6 and 24 h after injection. All 12 patients received SPECT/CT scans (SPECT: 60 projections, 20 s each, 256 × 256 pixel matrix; CT: 130 kV, effective 36 mAs) at 2 h and only SPECT scans (60 projections, 20 s each, 256 × 256 pixel matrix) 4 and 6 h after injection. For SPECT reconstruction, the xSPECT (Siemens) protocol based on the ordered subset conjugate gradient (OSCG) method (24 iterations, 2 subsets) was used. The 3D Gaussian FWHM 10 mm filter (Soft Tissue) was used. For processing of obtained images, the proprietary software package syngo.via (Siemens) was used.

Vital signs were monitored before, during and after imaging. Parameters of blood biochemistry were analyzed before injections of [^99m^Tc]Tc-TG and 24, 48 h and 7 days after injections.

### 2.4. Evaluation of Distribution and Dosimetry

Regions of interest (ROI) were drawn over organs of interest and the body contour on the anterior and posterior whole-body images. A geometric mean of counts at 2, 4, 6 and 24 h was calculated for each ROI. A known activity of ^99m^Tc in a water-filled phantom was measured and used for quantification. Chang’s correction was used for attenuation correction. To assess the activity in the blood, the data from a POI placed over the heart were used. Data were fitted to single exponential functions and residence times were calculated as areas under fitted curves using Prism 9 (version 9.3.1, GraphPad Software, LLC, San Diego, CA, USA). Absorbed doses were calculated by OLINDA/EXM 1.1 using an adult female and male phantoms.

To calculate tumor-to-background ratios at 2, 4 and 6 h, a one cm^3^ volume of interest (VOI) was drawn on tomograms centered on the highest tumor uptake, and counts were recorded. Thereafter, this VOI was copied to the contralateral side to obtain reference counts. The maximal standard uptake value (SUVmax) in nodal or extranodal lesions with the highest [^99m^Tc]Tc-TG accumulation was calculated 2 h after injection when CT data was available. Tumor sizes were defined by CT as the maximum size of the largest lesion.

### 2.5. Statistics

Values are reported as mean ± standard deviation. The significance of differences between uptakes in organs at different time points was analyzed using 1-way ANOVA. 

## 3. Results

### 3.1. Safety and Tolerability

The intravenous bolus administration of [^99m^Tc]Tc-TG was safe and well-tolerated by all patients. At all time points of objective control over the somatic state of patients after intravenous administration of [^99m^Tc]Tc-TG, no changes in vital signs or adverse reactions were registered. Patients did not actively present any complaints. The functional state of the main organs and systems did not have significant differences before and after the administration of [^99m^Tc]Tc-TG, which was confirmed by the results of the performed instrumental and laboratory tests. No changes in blood or urine samples were found (Table S1).

### 3.2. Evaluation of Distribution and Dosimetry

The kinetics of [^99m^Tc]Tc-TG elimination from blood is shown in Figure S2. The elimination half-lives were 3.1 h (95% CI 1.3 to 16 h) in female patients and 3.6 h (95% CI 1.6 to 19 h) in male patients. 

**Figure 2:**
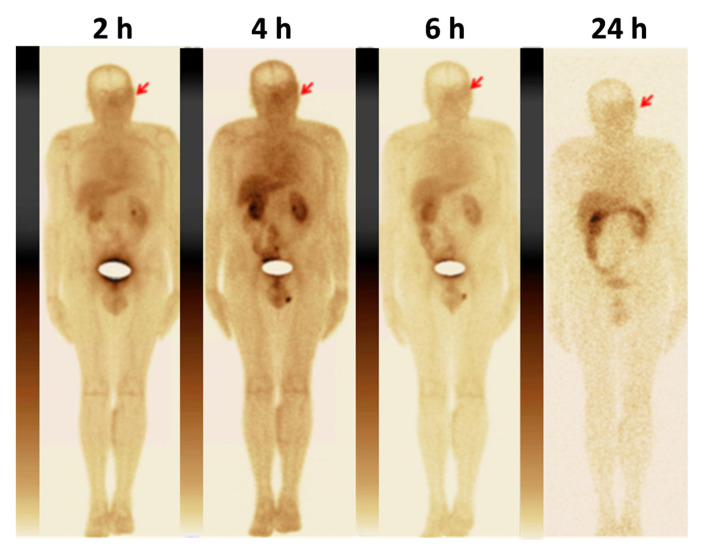
Anterior images of patient 7 (male) at 2, 4, 6 and 24 h after injection of [^99m^Tc]Tc-TG. Activity in the urinary bladder is masked. The upper setting of the scale window is 100% of the maximum counts.

The kidneys, liver and lungs were the organs with the highest accumulation of activity (Table 2; Figure 2). Noticeable activity was also observed in the gall bladder, spleen, thyroid, small intestines, testes and stomach. No significant difference between male and female patients was found.

The calculated absorbed doses is shown in Table 3. The highest absorbed dose was in the kidneys and urinary bladder wall, followed by the adrenals, prostate, osteogenic cells, lungs, heart wall, ovaries, uterus, liver and gall bladder wall. The effective doses were 0.0072 ± 0.0036 and 0.0135 ± 0.0091 mSv/MBq for female and male patients, respectively. For a typical injected activity of 730 MBq, which was used in this study, an expected effective dose would be 5.0–9.8 mSv.

### 3.3. [^99^^m^Tc]Tc-TG SPECT/CT Imaging of Nodal and Extranodal Lesions

**Figure 3:**
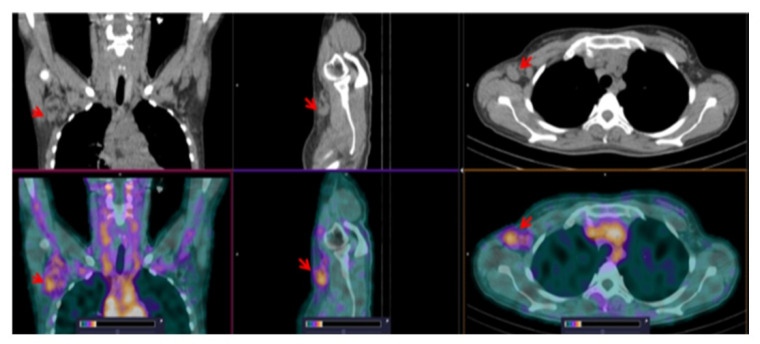
SPECT/CT images of patient 11 at 2 h after injection of [^99m^Tc]Tc-TG. An enlarged (up to 2.2 cm) right axillary node with elevated [^99m^Tc]Tc-TG uptake (SUVmax = 1.86) is visualized (arrows). The upper setting of the scale window (22% of the maximum number) was adjusted to visualize the lesion.

**Figure 4:**
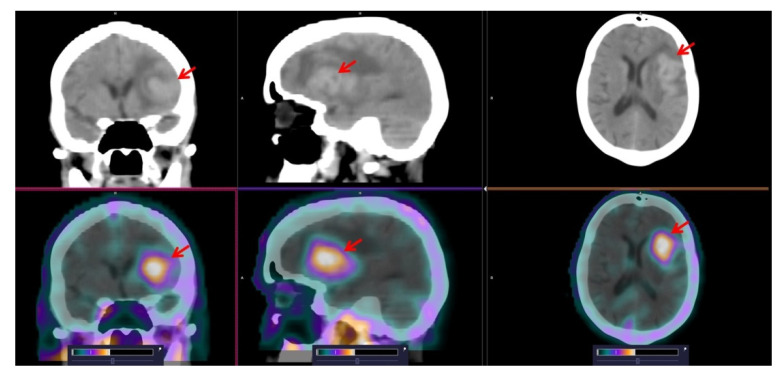
SPECT/CT images of patient 12 at 2 h after injection of [^99m^Tc]Tc-TG. High [^99m^Tc]Tc-TG uptake in left frontoparietal region of the brain (SUVmax = 2.65) is visualized (arrows). The upper setting of the scale window (65% of the maximum number) was adjusted to visualize the lesion.

**Figure 5:**
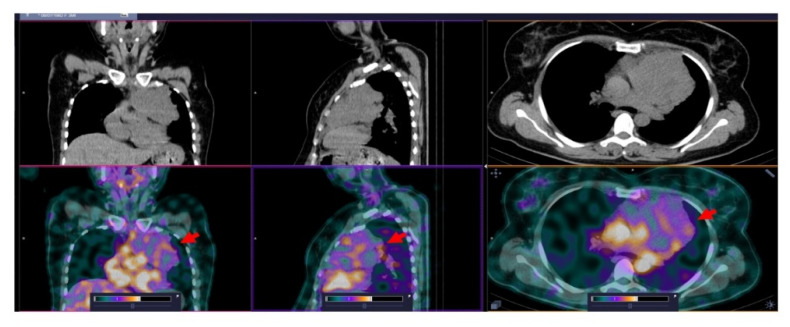
SPECT/CT images of patient 3 at 2 h after injection of [^99m^Tc]Tc-TG. Elevated [^99m^Tc]Tc-TG uptake in the mediastinal tumor (SUVmax = 1.3) is visualized (arrows). The upper setting of the scale window (60% of the maximum number) was adjusted to visualize the lesion.

**Figure 6:**
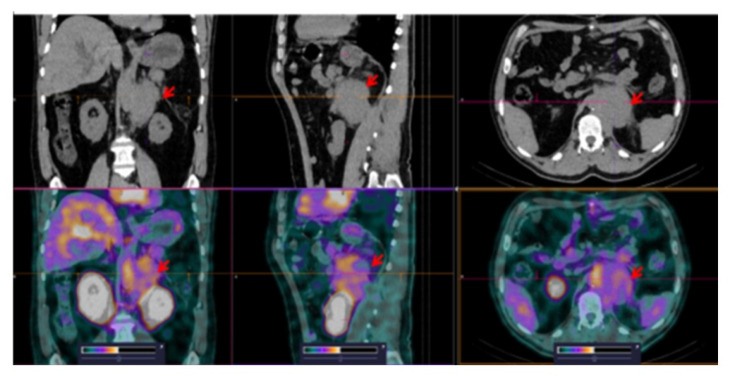
SPECT/CT images of patient 1 at 2 h after injection of [^99m^Tc]Tc-TG. Elevated [^99m^Tc]Tc-TG uptake in the retroperitoneal tumor (SUVmax = 3.92) is visualized (arrows). The upper setting of the scale window (40% of the maximum number) was adjusted to visualize the lesion.

**Figure 7:**
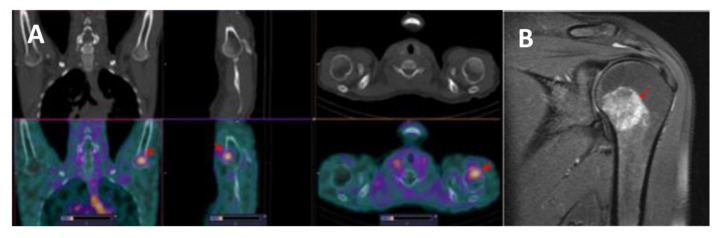
(**A**) SPECT/CT images of patient 11 at 2 h after injection of [^99m^Tc]Tc-TG. Elevated [^99m^Tc]Tc-TG uptake in the humerus head (SUVmax = 2.28) is visualized (arrows). No pathological changes were found using CT. The upper setting of the scale window (22% of the maximum number) was adjusted to visualize the lesion. (**B**) MRI image of the left shoulder joint, T1.

According to standard diagnostic methods (clinical examination, CT and ultrasound), lymph node lesions were known in 9 of the 12 examined lymphoma cases and extranodal lesions also in 9 of the patients. The [^99m^Tc]Tc-TG SPECT/CT scans revealed foci of elevated radiopharmaceutical uptake in the lymph nodes of all nine patients with nodal lesions (example in Figure 3). Extranodal lesions were also detected by [^99m^Tc]Tc-TG SPECT in all nine of the known cases. [^99m^Tc]Tc-TG SPECT/CT correctly visualized brain (Figure 4), neck, chest (Figure 5) and abdominal lesions (Figure 6). In one patient, according to [^99m^Tc]Tc-TG SPECT/CT, a lesion in the humerus head was visualized, which was not detected by CT (Figure 7A). Based on this finding, an MRI of the left shoulder joint was performed and a lymphoma lesion in the metaepiphysis of the humerus was confirmed (Figure 7B) and the stage of the disease was changed from IIIB to IVB. In the highest [^99m^Tc]Tc-TG accumulating lesions, SUVmax was 2.6 ± 1.1 at 2 h after injection (Table 4). The tumor/contralateral background ratios for extracranial lesions reached 3.3 ± 1.2 at 2 h after injection, decreased to 2.3 ± 1.4 after 4 h and did not change significantly after that (Table 4).

**Figure 8:**
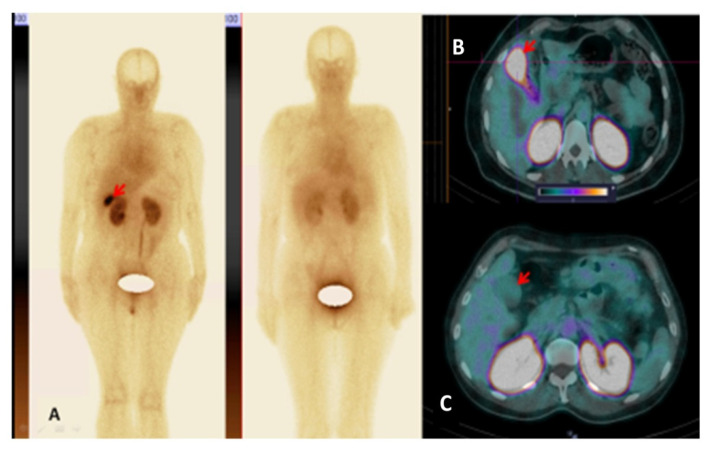
(**A**) Anterior images of female patient 5 (left) and female patient 4 (right) at 2 h after injection of [^99m^Tc]Tc-TG. High [^99m^Tc]Tc-TG uptake in the gallbladder in patient 5 is visualized (arrow). The upper setting of the scale window is 100 % of the maximum counts. (**B**) SPECT/CT image of patient 5 at 2 h after injection of [^99m^Tc]Tc-TG. High [^99m^Tc]Tc-TG uptake (SUVmax = 20.89) in the gallbladder in Patient 5 is visualized (arrow). (**C**) SPECT/CT image of patient 4 at 2 h after injection. There is no pathological uptake in the gallbladder of patient 4 (SUVmax = 2.87) (arrow). The upper setting of the scale window (100% of the maximum number).

In our study, patient 5 had cholecystitis as a concomitant diagnosis. In this case, a high [^99m^Tc]Tc-TG uptake (SUVmax = 20.8 vs. 4.4 ± 1.6 in normal patients) in the gallbladder was found (Figure 8, Table 4). In the rest of the patients, elevated [^99m^Tc]Tc-TG accumulation in the gallbladder was not visualized (Figure 8, Table 4).

## 4. Discussion

This study demonstrated that the injection of [^99m^Tc]Tc-TG is well-tolerated and not associated with any adverse effects. The favorable dosimetry properties of ^99m^Tc ensured a moderately effective dose. In the current study, typical equivalent doses were 5.0–9.8 mSv. However, the injected activity in this study was specifically selected to obtain good counting statistics 24 h after injection for dosimetry calculations. For routine use, the injected activity could be reduced at least three-fold (see below) with a proportional reduction in the effective dose. For comparison, a typical effective dose from [^18^F]-FDG PET is 3.5 mSv when 185 MBq is administered [13]. 

[^99m^Tc]Tc-TG SPECT allowed us to identify foci corresponding to all nodal lesions found according to the standard diagnostic methods in this study. There was also an excellent correlation between visualizing extranodal lesions using [^99m^Tc]Tc-TG SPECT and the standard diagnostic methods in all cases of brain (Figure 4), neck, chest (Figure 5) and abdominal lesions (Figure 6). A major advantage of [^18^F]-FDG PET/CT over CT is the possibility to detect bone marrow lesions in lymphomas, since CT allows visualization only in the case of bone destruction [14]. The use of [^99m^Tc]Tc-TG SPECT enabled detection of a bone lesion, which was not visualized by CT (Figure 7A). This finding prompted an additional MRI examination, which confirmed a lymphoma lesion in the humerus (Figure 7B). For this patient, the stage of the disease was changed based on [^99m^Tc]Tc-TG SPECT imaging followed by MRI confirmation. 

Because the tumor-to-background ratio reached 3.3 ± 1.2 at 2 h after injection, then decreased to 2.30 ± 1.42 after 4 h and after that did not change significantly, the optimal time for SPECT imaging is 2 h after the injection. At this time point, the [^99m^Tc]Tc-TG SUVmax was 2.6 ± 1.1, which is considerably lower than the typical [^18^F]-FDG SUVmax in lymphoma lesions according to the literature [15]. A possible explanation for this is that [^99m^Tc]Tc-TG is not internalized after binding to GLUT1 to the same extent as [^18^F]-FDG [4] and does not undergo metabolic trapping. Since [^99m^Tc]Tc-TG images likely GLUT-receptor expression rather than glucose metabolism, it will be essential for future clinical applications to confirm that the uptake of [^99m^Tc]Tc-TG in the rest of the human body is also GLUT-mediated. It is well known that GLUT receptors are overexpressed in inflammatory cells leading to an active uptake of [^18^F]-FDG in inflammatory foci [16]. In particular, an accumulation of [^18^F]-FDG in cholecystitis has been previously demonstrated [17,18,19]. For the study herein, imaging of patient 4 with documented chronic cholecystitis showed a much higher activity accumulation in the gallbladder, compared to other patients (Table 4; Figure 8). This suggests that [^99m^Tc]Tc-TG uptake is dependent on GLUT-receptor expression, similar to [^18^F]-FDG, which might support its use for the imaging of hypermetabolic lesions overexpressing these receptors. Unfortunately, this also means that all pitfalls and artifacts known when using [^18^F]-FDG for the imaging of hematologic malignancies [20] might also apply when using [^99m^Tc]Tc-TG. 

Currently, [^18^F]-FDG PET is applied for post-therapy-response assessment in “[^18^F]-FDG-avid” lymphomas [21,22]. Investigating the use of [^99m^Tc]Tc-TG for this application might also be worthwhile, taking into account that current SPECT/CT technology permits the semiquantitative assessment of uptake.

Another interesting finding is that [^99m^Tc]Tc-TG is not accumulated in the brain, despite GLUT1 being the main transporter for glucose through the intact blood–brain barrier [23]. This might be because the transport of [^99m^Tc]Tc-TG through cellular membranes is impaired [4]. This phenomenon might facilitate the use of [^99m^Tc]Tc-TG for the visualization of lymphoma-associated brain lesions (Figure 4) and brain tumors, where the blood–brain-barrier is typically disrupted. 

Obviously, [^99m^Tc]Tc-TG SPECT/CT cannot compete with [^18^F]-FDG PET/CT in countries where PET is available. First, the accumulation of [^18^F]-FDG in lymphoma lesions is higher, which is a prerequisite for better imaging contrast. Second, sensitivity and quantification accuracy of PET is better. Still, [^99m^Tc]Tc-TG might be the only tracer for molecular imaging of lymphoma for patients living in countries with poor or no access to PET. Therefore, we believe that further clinical development of this tracer is warranted.

## 5. Conclusions

Injections of [^99m^Tc]Tc-TG appear safe and the radiation burden was comparable to the burden from [^18^F]-FDG. The [^99m^Tc]Tc-TG showed an elevated uptake in the nodal and extranodal lesions in malignant lymphomas patients. Further studies concerning the use of [^99m^Tc]Tc-TG SPECT/CT for lymphoma staging and therapy monitoring are justified.

## Figures and Tables

**Table 1:** Initial status of the patients recruited for the clinical study.

Patient No	Sex	Age (y)	IHC Diagnosis	Stage
1	Male	62	FL	IVA
2	Male	39	HL	IVA
3	Female	20	HL	IIIB
4	Female	57	DLBCL	IVB
5	Female	49	MALT-lymphoma	IIIB
6	Female	56	BLL	IVA
7	Male	58	FL	IVA
8	Female	45	HL	IIA
9	Male	68	DLBCL	IVA
10	Female	39	MZL	IVA
11	Female	34	HL	IIIB *
12	Female	61	DLBCL	IVA

FL—Follicular lymphoma; HL—Hodgkin lymphoma; DLBCL—Diffuse large B-cell lymphoma; MALT-lymphoma—mucosa-associated lymphoid tissue lymphoma; BLL—B-lymphoblastic lymphoma; MZL—Marginal zone lymphoma. *—stage was changed to IVB based on [^99m^Tc]Tc-TG SPECT/CT and additional MRI studies performed after [^99m^Tc]Tc-TG SPECT/CT.

**Table 2:** Decay-corrected uptake of ^99m^Tc in the highest-uptake organs based on planar imaging of tumor-free areas. The data are presented as average %ID ± SD per organ for six females and four males at different time points after injection with [^99m^Tc]Tc-TG.

Time	Kidney		Liver		Lung	
	Female	Male	Female	Male	Female	Male
2 h	5.6 ± 2.7	4.0 ± 0.7	3.5 ± 1.1	3.4 ± 1.0	4.0 ± 1.5	4.6 ± 1.1
4 h	6.5 ± 3.5	4.3 ± 0.9	3.6 ± 0.8	3.2 ± 0.8	4.5 ± 2.1	4.0 ± 0.9
6 h	5.9 ± 3.1	3.7 ± 1.3	3.9 ± 1.2	2.5 ± 0.8	3.7 ± 1.2	3.1 ± 1.1
24 h	7.1 ± 3.7	4.5 ± 2.1	2.9 ± 1.1	2.6 ± 0.5	2.7 ± 1.0	2.5 ± 0.5

**Table 3:** Absorbed doses (mGy/MBq) after injection of [^99m^Tc]Tc-TG.

Site	Female Patients	Male Patients
Testes		0.01 ± 0.01
Brain	0.0017 ± 0.0006	0.0012 ± 0.0003
Breasts	0.002 ± 0.001	0.0011 ± 0.0002
Skin	0.0019 ± 0.0007	0.0014 ± 0.0003
Muscle	0.003 ± 0.001	0.0021 ± 0.0005
Red Marrow	0.003 ± 0.001	0.0026 ± 0.0005
Stomach Wall	0.004 ± 0.001	0.0028 ± 0.0005
Small Intestine	0.005 ± 0.002	0.0042 ± 0.0009
LLI Wall	0.005 ± 0.003	0.005 ± 0.003
Thymus	0.005 ± 0.002	0.005 ± 0.001
Thyroid	0.005 ± 0.002	0.004 ± 0.001
Spleen	0.005 ± 0.002	0.0032 ± 0.0006
Pancreas	0.005 ± 0.002	0.0033 ± 0.0004
ULI Wall	0.005 ± 0.002	0.0038 ± 0.0008
Gallbladder Wall	0.007 ± 0.003	0.005 ± 0.001
Liver	0.007 ± 0.002	0.004 ± 0.001
Uterus	0.007 ± 0.004	
Ovaries	0.007 ± 0.04	
Heart Wall	0.008 ± 0.003	0.006 ± 0.002
Lungs	0.008 ± 0.003	0.005 ± 0.001
Osteogenic Cells	0.008 ± 0.003	0.006 ± 0.001
Adrenals	0.009 ± 0.003	0.005 ± 0.001
Kidneys	0.03 ± 0.01	0.02 ± 0.01
Urinary Bladder Wall	0.03 ± 0.04	0.05 ± 0.05
Prostate		0.010 ± 0.006
Total Body	0.004 ± 0.002	0.0028 ± 0.0005
Effective Dose Equivalent (mSv/MBq)	0.009 ± 0.005	0.02 ± 0.01
Effective Dose (mSv/MBq)	0.007 ± 0.004	0.014 ± 0.009

**Table 4:** Tumor size, SUVmax (at 2 h) and tumor-to-contralateral site ratios (at 2, 4 and 6 h) after the injections of [^99m^Tc]Tc-TG.

Patients	Tumor Size cm	SUVmax Tumor	SUVmax Gallbladder	Tumor/ Background 2 h	Tumor/ Background 4 h	Tumor/ Background 6 h
1.	8.0	3.92	4.2	4.1	4.2	2.7
2.	1.8	2.23	7.1	2.5	1.6	1.4
3.	12.0	1.3	-	2.5	1.6	1.8
4.	3.8	4.53	2.9	1.7	1.9	1.9
5.	3.6	3.39	20.9	3.5	1.4	1.3
6.	4.6	3.34	4.2	3.6	1.6	1.7
7.	5.0	2.45	-	2.5	1.7	1.6
8.	4.5	1.43	-	3.8	2.5	2.8
9.	2.8	1.89	-	2.8	1.3	1.48
10.	8.3	1.21	-	2.9	1.7	3.2
11.	4.2	2.28	3.5	6.3	5.8	4.2
12.	4.6	2.65	-	21.9	18.3	16.1
Mean ± SD	5 ± 3	3 ± 1	4.4 ± 1.6 *	3.3 ± 1.2 **	2.3 ± 1.4 ** p = 0.005 ***	2.1 ± 0.9 ** p = 0.005 ***

*—Mean ± SD was calculated for normal [^99m^Tc]Tc-TG gallbladder uptake, ** values for extracranial lesions; *** the significance of the differences is shown in comparison with the tumor/background value for extracranial lesions at 2 h.

## Data Availability

Data is contained within the article.

## Supplementary Materials

The following supporting information can be downloaded at: https://www.mdpi.com/article/10.3390/pharmaceutics14061274/s1, Figure S1: Standards for Reporting of Diagnostic Accuracy Studies (STARD) flow diagram; Figure S2: Kinetics of elimination of [^99m^Tc]Tc-TG from blood. Data are based on count rates in regions of interest placed over hearts. %ID = percentage injected dose; Table S1: Parameters of blood biochemistry in patients before intravenous administration of a radiopharmaceutical and after 24, 48 h and 7 days post administration.
