# Supplementary material for: Phase I Clinical Trial Using [99mTc]Tc-1-thio-D-glucose for Diagnosis of Lymphoma Patients
Source: Pharmaceutics. 2022 Jun 15;14(6):1274. doi: 10.3390/pharmaceutics14061274 (PMC9227866; doi:10.3390/pharmaceutics14061274)
Supplement: Supplementary file 1 [file pharmaceutics-14-01274-s001.zip › pharmaceutics-1727584-supplementary.pdf]

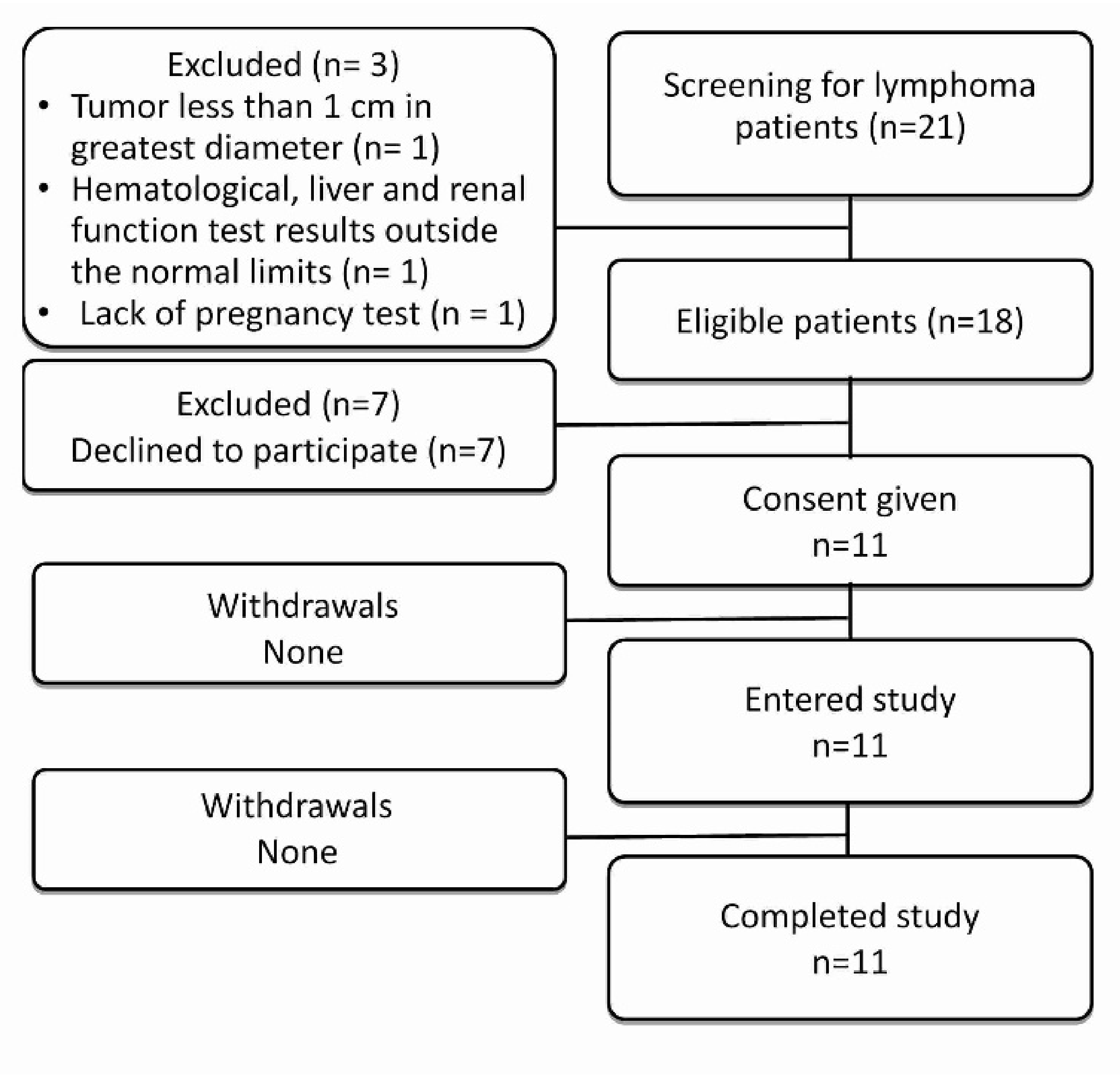

Figure S1. Standards for Reporting of Diagnostic Accuracy Studies (STARD) flow diagram.

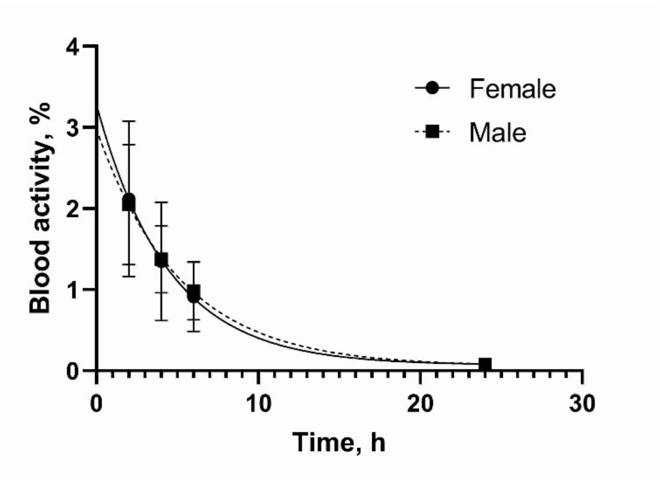

Figure S2. Kinetics of elimination of ^{99m}Tc-TG from blood. Data are based on count rates in regions of interest placed over hearts. %ID = percentage injected dose.

Table S1. Parameters of blood biochemistry in patients before intravenous administration of a radiopharmaceutical and after 24, 48 hours and 7 days post administration.

Blood index	Before intravenous administration Me (Q1–Q3)	After a single intravenous administration		
		24 h Me (Q1–Q3)	48 h Me (Q1–Q3)	7 days Me (Q1–Q3)
Alanine aminotransferase, U/L	13.0 (12–15)	14.0 (11–16) $p = 0.25$	13.0 (12–15) $p = 0.26$	13.0 (11–16) $p = 0.25$
Aspartate aminotransferase, U/L	14.0 (12–16)	15.0 (12–17) $p = 0.31$	14.0 (12–16) $p = 0.32$	14.5 (13–16) $p = 0.29$
Alkaline phosphatase, U/L	162.0 (145–200)	161.0 (143–200) $p = 0.21$	162.5 (151–205) $p = 0.25$	162.5 (151–206) $p = 0.24$
Glucose, mmol/L	4.5 (4.2–5.6)	4.45 (4.2–5.4) $p = 0.21$	4.45 (4.1–5.4) $p = 0.22$	4.5 (4.1–5.4) $p = 0.25$
Creatinine, µmol/L	84.0 (66–89)	85.0 (68–92) $p = 0.24$	84.0 (66–90) $p = 0.28$	85.0 (68–93) $p = 0.31$
Protein, g/L	74.0 (64–75)	73.5 (66–74) $p = 0.31$	73.5 (65–75) $p = 0.24$	74.5 (67–77) $p = 0.29$
Bilirubin, µmol/L	9.7 (6.0–12.5)	9.8 (6.5–12.6) $p = 0.22$	9.9 (6.2–12.5) $p = 0.33$	9.8 (5.6–12.9) $p = 0.36$
